# Heroin-Induced Leukoencephalopathy: A Case Report

**DOI:** 10.7759/cureus.68110

**Published:** 2024-08-29

**Authors:** Daniel I Casal, Asya Wallach

**Affiliations:** 1 Psychiatry and Behavioral Sciences, Neurology, Rowan-Virtua SOM (School of Osteopathic Medicine), Stratford, USA; 2 Multiple Sclerosis, Neurology, Holy Name Hospital, Teaneck, USA

**Keywords:** physical medicine & rehabilitation, white matter hyperintensity, reversible dementia, flair, heroin inhalation, toxic leukoencephalopathy, heroin-induced leukoencephalopathy

## Abstract

Heroin-induced leukoencephalopathy (HLE) is a rare disease that can present with a variety of neurological symptoms ranging from mild to severe, including death. This condition is associated with inhaling heroin, a phenomenon well-documented in the literature and termed “chasing the dragon”. Here, we discuss a case of a 27-year-old female who presented with subacute neurological symptoms following two years of recreational heroin inhalation. Magnetic resonance imaging (MRI) findings were consistent with diffuse symmetrical corticospinal tract hyperintensities. Herein, we will describe her clinical course with one year of follow-up.

## Introduction

Toxic leukoencephalopathy is a neurological disorder in which the white matter of the brain is damaged by a leukotoxic substance. Toxic leukoencephalopathy may occur due to exposure to drugs of abuse (cocaine, heroin), chemotherapeutic agents (methotrexate and 5-fluorouracil), and environmental toxins [1]. Various modes of heroin consumption have been reported to cause toxic leukoencephalopathy, including inhalation and subconjunctival heroin injections. “Chasing the dragon” is an inhalation mode of heroin abuse that avoids IV-associated complications and involves heating the opioid in an aluminum foil above a flame [2]. This creates the illusion of inhaling the fumes of the dragon's tail. This mode of drug use has been reported to have originated in Southeast Asia hundreds of years ago [2]. However, heroin-induced leukoencephalopathy (HLE) has been first documented occurring in the Netherlands and is specifically associated with inhaling heroin [2].

There are a lot of clinical manifestations associated with leukoencephalopathy, including dementia, consciousness impairment, cognitive dysfunction, ataxia, dysarthria, motor weakness, and even death [1]. The timeframe for symptom presentation depends on how long the patient has been using the substance. Some patients have reportedly presented with acute symptoms immediately after using a toxic substance. However, the factors that can predispose an individual to an acute or chronic presentation, as well as the severity of the presentation are unknown [3]. Brain magnetic resonance imaging (MRI) typically reveals a posterior predominant (cerebellum, posterior cerebrum, or posterior internal capsule), symmetric hyperintense T2/fluid-attenuated inversion recovery (FLAIR) signal changes. Biopsy specimens taken from autopsies of affected individuals reveal extensive spongiform degeneration and even vacuole formation of the cerebral white matter [4].

Here, we present an atypical case of HLE occurring in a 27-year-old female with a history of heroin use who presented with severe neurological symptoms. This case is unique because even though the patient presented with debilitating symptoms after heroin use, she recovered dramatically with minimal neurological symptoms noted on the neurological exam at five months, highlighting a possible positive outcome.

## Case presentation

**Figure 1:**
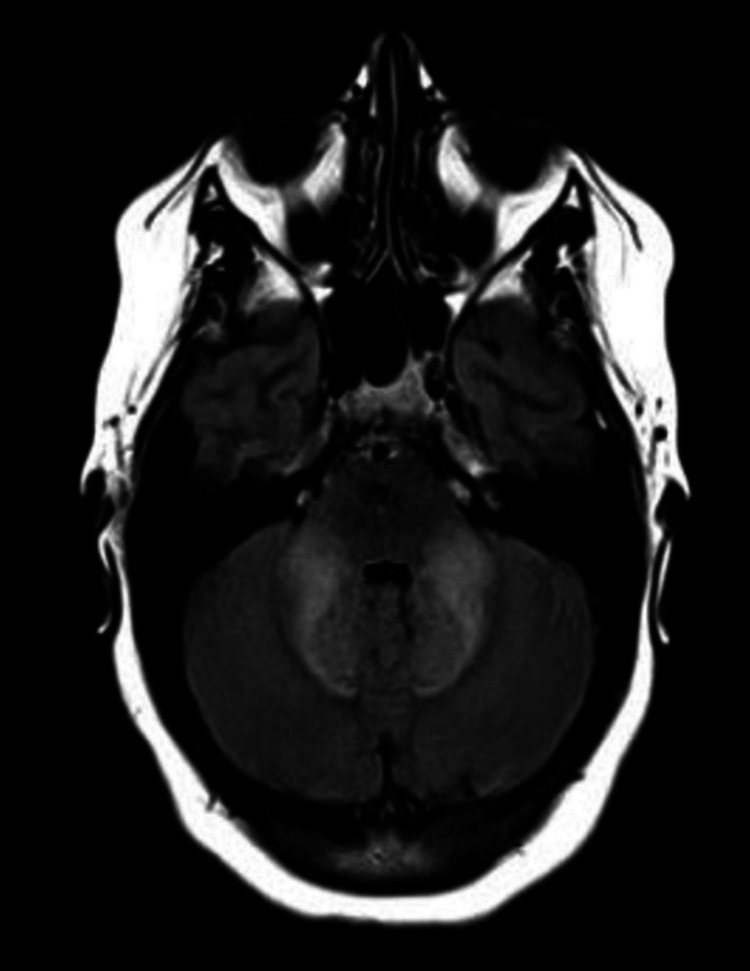
FLAIR image reflecting the symmetry and extent of the hyperintensities in the brachium pontis FLAIR: fluid-attenuated inversion recovery

**Figure 2:**
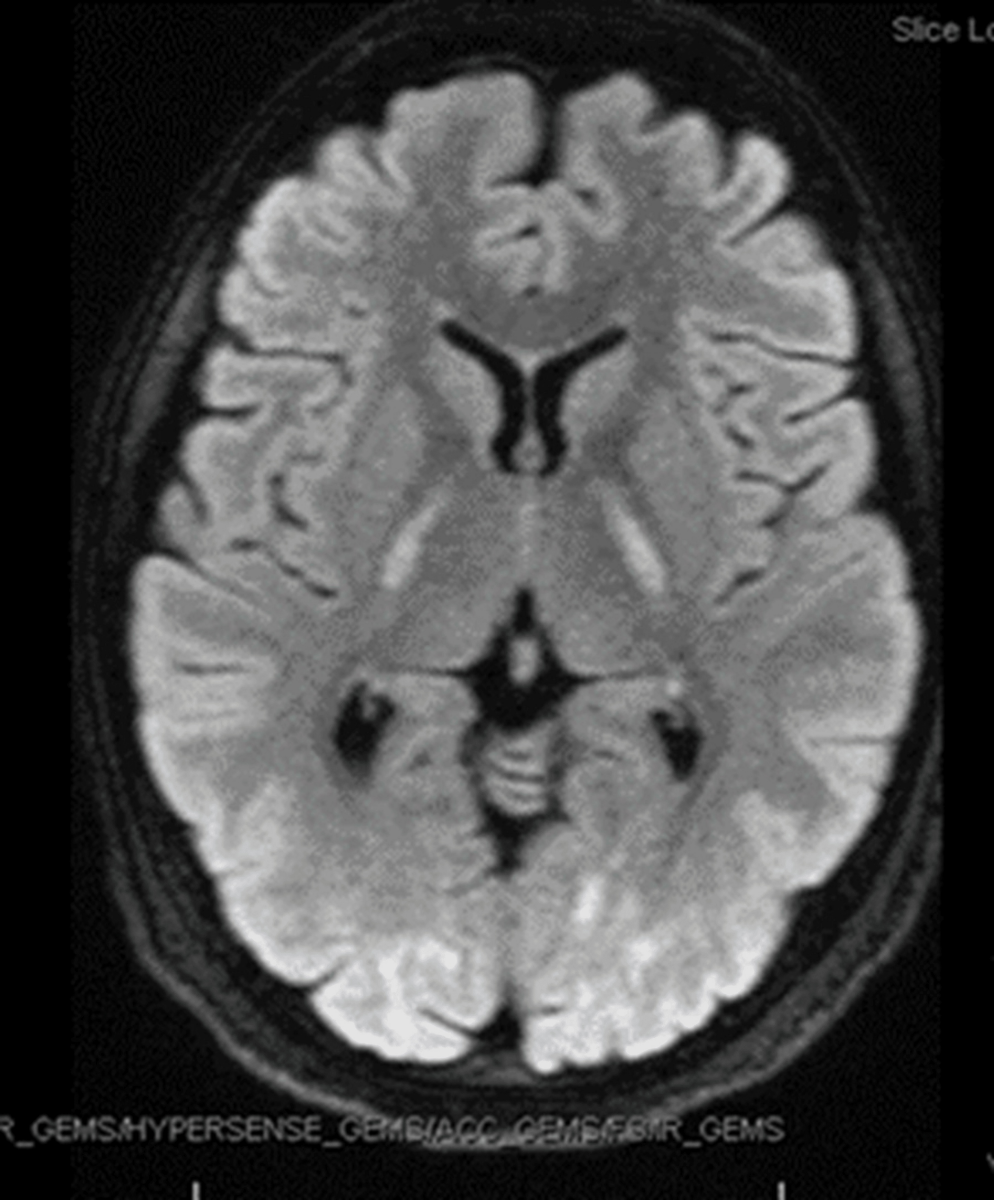
Flair axial view showing hyperintensities in the occipital lobe and the internal capsule FLAIR: fluid-attenuated inversion recovery

A 27-year-old female with a past medical history of migraines and substance use disorder (heroin and marijuana) presented for the evaluation of acute-onset dysgraphia, dysarthria, and acalculia. A CT scan of the head performed at an outside institution revealed abnormalities, however, the patient left and did not follow up with care. A week later, she was admitted to our hospital for heroin withdrawal and difficulty ambulating and left against medical advice shortly after. Two weeks later, she presented to a third hospital, with worsened ambulation, requiring a wheelchair. She also had difficulty doing arithmetic calculations, speech impairment, severe fatigue, memory loss, and coordination impairment. Serum antibody testing was negative for neuromyelitis optica (NMO) and myelin oligodendrocyte glycoprotein antibody disease (MOGAD). Serum tests for vitamin B1, angiotensin-converting enzyme (ACE), venereal disease research laboratory test (VDRL), Lyme, hepatitis screen, alcohol level, acetaminophen, and salicylate levels were unremarkable. Aspartate aminotransferase (AST) and alanine aminotransferase (ALT) levels were within normal limits. FLAIR images revealed symmetrical hyperintensities in the brachium pontis (Figure 1). Other notable FLAIR imaging findings included hyperintensities in the occipital lobe and the internal capsule (Figure 2). MRI of the cervical and thoracic spine revealed no abnormal findings. Given her history of heroin use and neurological and imaging findings, she was diagnosed with HLE and discharged to an acute rehabilitation hospital where she was treated with physical therapy, speech therapy, and occupational therapy, in addition to escitalopram 10 mg, hydroxyzine 25 mg, coenzyme Q10, vitamin C, gabapentin, and Vitamin E. She discontinued the medications at the time of her discharge.

A follow-up visit five months later, the patient had significant ambulatory recovery; she did not need any assistive device at the time of the office visit but acknowledged that sometimes she needs assistance when walking. She reported balancing difficulties occasionally, requiring her to look down at her feet while walking. She recovered from her heroin withdrawal and has not relapsed since the previous admission. Physical exam showed the patient was able to speak fluently, without any slurring or trouble pronouncing words, and the mini-mental status exam was normal. She had mild, proximal, left-sided weakness at the left deltoid and hip flexor; otherwise, she had full strength, sensation, coordination, and symmetric normal deep tendon reflexes. Her gait was unremarkable, including intact tandem and negative Romberg.

## Discussion

HLE was first documented in Europe, with more cases reported across the globe [2]. Other leukotoxins have also been associated with affecting white matter tracts causing a wide variety of clinical symptoms. Cases of TLE may be underreported mainly because TLE can be associated with drugs of abuse, causing overdose and subsequent death before diagnosis can be made. The exact pathophysiological mechanism of TLE is not well understood; however, several hypotheses have tried to explain the mechanism of the condition. The white matter damage may be from direct toxic injury on the myelin sheath or it can be indirectly from capillary endothelial injury or a combination of both [5]. However, it may be possible that in heroin-related cases of leukoencephalopathy, there can be an unknown contaminant that varies by region, distribution, and production of the drug that can be responsible for the white matter changes seen in the condition [6].

Clinically, symptoms can vary on a case-by-case basis; however, patients reportedly progress through three stages lasting from weeks to months. The first stage of the disease typically involves cerebellar dysfunction, including difficulty with speech, ataxia, and motor restlessness. The second stage usually involves myoclonic jerks, choreoathetoid movements, and spastic paresis. The third stage includes akinetic mutism, extensor posturing, pyrexia, and eventually death [7]. Depending on which areas of the brain are affected, different clinical features of the disease may be expressed. Other notable symptoms include personality changes, dysarthria, forgetfulness, inattention, dementia, coma, and even death [8]. Our patient presented various neurological findings. Her most prominent symptoms included memory loss, impaired movement and coordination, fatigue, and speech impairment.

Typical T2-weighted MRI findings reported in the literature that differentiate heroin-induced leukoencephalopathy (HIL) from other causes of leukoencephalopathy include bilateral symmetric damage to cerebellar white matter, posterior cerebral white matter, such as the occipital lobe, cerebellar peduncles, and the posterior limb of the internal capsule [9]. MRI findings help confirm the diagnosis; however, other modalities have also been used to support the diagnosis. Studies have shown that proton magnetic resonance spectroscopy (H MRS) has helped establish the diagnosis of progressive multifocal leukoencephalopathy (PML) and other variants of leukoencephalopathy. Prominent peaks obtained in H MRS for a study was lactate, which showed a nearly 3 times higher concentration of lactate compared to choline, a 4-6 times higher lactate compared to creatine concentration, and 4-11 times higher lactate compared to N-acetylaspartate concentration. This pattern seemed to be consistent and observed in the three patients of the study, indicating this tool may be useful in confirming an early diagnosis of PML [10]. Another case report using HMRS on a HIL patient noted a decrease in the N-acetylaspartate/creatine ratio (NAA/Cr) and a doublet lactate peak, which indicates mitochondrial dysfunction and neurotoxicity [11]. MRI is useful in precisely revealing the distribution of white matter abnormalities in patients with HIL, and H MRS is another modality that can be used to help further confirm the diagnosis. However, further studies need to be conducted to prove the usefulness of H MRS in patients with HIL.

Treatment for TLE should involve targeting reversible causes, prevention, and symptom management [12]. Patients do seem to recover when the leukotoxin has been removed. In this patient, neurological symptoms rapidly worsened when the patient returned to the hospital after she reportedly was undergoing opioid withdrawal. Delayed apoptosis of oligodendrocytes may explain the clinical course of this patient. This concept has been reported in the literature describing several conditions such as carbon monoxide poisoning. It is believed that the toxic radicals accumulate over the oligodendrocytes over a period of time since exposure to the toxin [13]. The spontaneous recovery of the patient can best be explained by the gradual regeneration of the white matter. Additionally, certain medications have been thought to help in neurological recovery such as certain vitamins. Vitamins E and C have antioxidant properties that can help increase the secretion of neurotrophic cytokines and later improve nerve states [14].

Our patient seemed to have benefited from antioxidant therapy, physical therapy, and rehabilitation. The patient's unexpected and near complete recovery on neurological examination differs from other case report findings. However, this may suggest that there are other parameters involved in the course of this condition. Patients may fully recover depending on the extent of white matter damage seen on imaging, however, this topic remains undetermined. Other factors to consider include the patient's history of drug use, the quantity of the drug consumed during a period of time, the type of treatment regimen the patient followed, and other external factors. Despite the initial poor presentation of neurological symptoms, it seems that recovery is still possible. However, more extensive studies need to be conducted with a greater sample size of patients and proper treatment protocols need to be found.

## Conclusions

HLE is a debilitating neurological condition with an unknown pathophysiology accompanied by various neurological symptoms. It is mostly seen in patients who inhale the vapor of heated heroin, however, it can also be seen in patients who administer heroin via other routes. The impaired neurological signs are almost always severe and deadly, with slow recovery. Brain imaging typically reveals diffuse symmetrical white matter changes. There is currently no Food and Drug Administration (FDA)-approved pharmacological intervention to treat HLE. Other interventions, such as physical therapy, antioxidants, and vitamins, have shown improvement. Interventions need to be focused on prevention and increasing awareness of the harmful effects of heroin abuse given the lack of treatment options and the extensive neurological deterioration associated with heroin abuse.

## References

[1] Filley CM, Kleinschmidt-DeMasters BK (2001). Toxic leukoencephalopathy. N Engl J Med.

[2] Bartlett E, Mikulis DJ (2005). Chasing "chasing the dragon" with MRI: leukoencephalopathy in drug abuse. Br J Radiol.

[3] Alambyan V, Pace J, Miller B (2018). The emerging role of inhaled heroin in the opioid epidemic: a review. JAMA Neurol.

[4] Alshamam MS, Sumbly V, Nso N, Saliaj M, Gurung DO (2021). Heroin-induced leukoencephalopathy. Cureus.

[5] Sharma P, Eesa M, Scott JN (2009). Toxic and acquired metabolic encephalopathies: MRI appearance. AJR Am J Roentgenol.

[6] Salgado RA, Jorens PG, Baar I, Cras P, Hans G, Parizel PM (2010). Methadone-induced toxic leukoencephalopathy: MR imaging and MR proton spectroscopy findings. AJNR Am J Neuroradiol.

[7] Al-Chalabi M, Lateef S, Gharaibeh K, Saraiya P, Ghannam M (2020). Mimicking a psychiatric disorder: heroin-induced leukoencephalopathy. Cureus.

[8] Buxton JA, Sebastian R, Clearsky L, Angus N, Shah L, Lem M, Spacey SD (2011). Chasing the dragon - characterizing cases of leukoencephalopathy associated with heroin inhalation in British Columbia. Harm Reduct J.

[9] Kass-Hout T, Kass-Hout O, Darkhabani MZ, Mokin M, Mehta B, Radovic V (2011). "Chasing the dragon"--heroin-associated spongiform leukoencephalopathy. J Med Toxicol.

[10] Kriegstein AR, Shungu DC, Millar WS (1999). Leukoencephalopathy and raised brain lactate from heroin vapor inhalation ("chasing the dragon"). Neurology.

[11] Kozić D, Bjelan M, Boban J (2017). A prominent lactate peak as a potential key magnetic resonance spectroscopy (MRS) feature of progressive multifocal leukoencephalopathy (PML): spectrum pattern observed in three patients. Bosn J Basic Med Sci.

[12] Tan TP, Algra PR, Valk J, Wolters EC (1994). Toxic leukoencephalopathy after inhalation of poisoned heroin: MR findings. AJNR Am J Neuroradiol.

[13] Offiah C, Hall E (2008). Heroin-induced leukoencephalopathy: characterization using MRI, diffusion-weighted imaging, and MR spectroscopy. Clin Radiol.

[14] Piantadosi CA, Zhang J, Levin ED, Folz RJ, Schmechel DE (1997). Apoptosis and delayed neuronal damage after carbon monoxide poisoning in the rat. Exp Neurol.

